# Comparing the broth enrichment-multiplex lateral flow immunochromatographic assay with real time quantitative PCR for the rapid detection of carbapenemase-producing organisms in rectal swabs

**DOI:** 10.1186/s12879-023-08244-6

**Published:** 2023-06-19

**Authors:** Yue Wang, Huijuan Song, Min Xu, Dengju Li, Xiao Ran, Ziyong Sun, Zhongju Chen

**Affiliations:** 1 Department of Laboratory Medicine, Tongji Hospital, Tongji Medical College, Huazhong University of Science and Technology, Wuhan, China; 2 Department of Infection Control and Prevention, Tongji Hospital, Tongji Medical College, Huazhong University of Science and Technology, Wuhan, China; 3 Department of Haematology, Tongji Hospital, Tongji Medical College, Huazhong University of Science and Technology, Wuhan, China; 4 Department of Emergency Medicine, Tongji Hospital, Tongji Medical College, Huazhong University of Science and Technology, Wuhan, China

**Keywords:** Immunochromatographic assay, Real-time quantitative polymerase chain reaction, NG-Test CARBA 5, Rectal swab, Carbapenemase-producing organism, Carbapenemase

## Abstract

**Background:**

Rapid and accurate identification of carbapenemase-producing organism (CPO) intestinal carriers is essential for infection prevention and control. Molecular diagnostic methods can produce results in as little as 1 h, but require special instrumentation and are expensive. Therefore, it is urgent to find an alternative method. The broth enrichment-multiplex lateral flow immunochromatographic assay was recently reported, but using it to directly detect CPO intestinal carriers in rectal swabs still requires the evaluation of many samples. The aim of this study was to compare the performance of these two methods, and to explore the control measures of CPO infection.

**Methods:**

Through CPO selective culture, PCR and DNA sequencing, 100 rectal swabs confirmed to be CPO-positive and 100 rectal swabs with negative results were collected continuously. After eluting the rectal swabs with saline, three aliquots were used: one for counting, one for detection by Xpert Carba-R, and one for culture in broth for 0 h, 1 h, 2 h, 3 h and 4 h, followed by NG-Test CARBA 5 assessment. The sensitivity and specificity of the NG-Test CARBA 5 method after different incubation times were calculated. The limit of detection (LoD) of this assay after 4 h broth incubation was estimated by examining the bacterial suspensions and simulated faecal suspensions prepared with CPOs producing different types of carbapenemases.

**Results:**

Xpert Carba-R demonstrated a combined sensitivity of 99.0% and specificity of 98.0%. The sensitivity and specificity were higher than 90.0% for the different enzyme types. The specificities of five common carbapenemases detected by the broth enrichment NG-Test CARBA 5 combined method after different incubation times were 100%. The sensitivities increased with increasing incubation time. At 4 h, the *Klebsiella pneumoniae* carbapenemase (KPC), New Delhi metallo-beta-lactamase (NDM), imipenemase (IMP), Verona integron-encoded metallo-beta-lactamase (VIM), and oxacillinase (OXA) -48 detection sensitivities were 93.0%, 96.3%, 100%, 100% and 85.7%, respectively. The LoDs were between 10^2^ and 10^4^ CFU/mL for all five enzymes after 4 h of incubation.

**Conclusions:**

This investigation highlighted that the broth enrichment-multiplex lateral flow immunochromatographic assay can be used as a new method for screening CPOs in rectal swabs.

**Supplementary Information:**

The online version contains supplementary material available at 10.1186/s12879-023-08244-6.

## Background

Carbapenemase-producing organisms (CPOs) are a group of bacteria with reduced sensitivity to carbapenems due to their production of carbapenemase. In an outbreak situation or endemic setting, information regarding CPO colonization status can potentially have important beneficial effects on empiric antibiotic treatment plans and infection prevention and control strategies [1]. Most CPOs contain one or more of the following five most prevalent carbapenemase families: *Klebsiella pneumoniae* carbapenemase (KPC), New Delhi metallo-beta-lactamase (NDM), imipenemase (IMP), Verona integron-encoded metallo-beta-lactamase (VIM), and oxacillinase (OXA) -48-like [2]. The identification of CPOs can be accelerated by rapid carbapenemase phenotype and gene detection methods [3–8]. However, most of these methods are used to detect bacterial colonies rather than specimens, so overnight culture is needed. Available molecular methods, such as Cepheid Xpert Carba-R and BD MAX Check-Points CPO assays, detect carbapenemase genes directly from clinical specimens and provide highly reliable results in a few hours [9, 10], but they are usually expensive and require special instrumentation. In addition, the real-time quantitative polymerase chain reaction (PCR) method is so sensitive that traces of DNA with antimicrobial resistance genes can be detected, even if they are devoid of a living CPO [11]. Therefore, it is urgent to find a rapid and cost-effective alternative for the large-scale screening of intestinal CPO carriers. NG-Test CARBA 5 (NG Biotech, Guipry, France) is a rapid, monoclonal antibody recognition visual redout multiplex lateral flow immunochromatographic assay method for use in vitro. It can simultaneously detect whether one or more of the above five carbapenemases are present in a single detection strip [12]. This assay has been proven to be effective in detecting bacterial colonies and positive blood cultures [13–15]. When using rectal swabs for direct detection, its detection sensitivity can be improved by incubation in broth for a few hours [16]. However, the methodological performance of the aforementioned two methods for CPO screening has not yet been compared. Therefore, a more comprehensive methodological evaluation with a larger sample size was designed and implemented in this study. This study aims to provide cost-effective solutions for CPO screening for infection prevention and control.

## Methods

### Collection of rectal swabs

This prospective study was conducted from September 2021 to December 2021 in the intensive care unit (52 beds) and haematology (215 beds) departments of a general teaching hospital in Wuhan, China. Anonymized samples that consisted of leftover rectal swabs from hospitalized patients taken for routine surveillance CPO screening were tested. A swab transport system (Copan Italia, Brescia, Italy) was used. CPO screening was performed by inoculating samples on CHROMagar KPC (CHROMagar, Paris, France). All coloured colonies obtained on the plate that appeared suspicious according to the manufacturer’s recommendations were subcultured for purity and then subjected to identification using an Autof ms1000 automatic microbial mass spectrometry detection system and to susceptibility testing for meropenem and imipenem using the Kirby-Bauer method. The interpretive criteria used followed the guidelines of the Clinical and Laboratory Standards Institute (CLSI) M100 Ed30 document [17]. Strains with inhibition zone diameters of less than 23 mm after treatment with meropenem or imipenem were collected. The carbapenemase production of these strains was phenotypically detected by a carbapenemase inhibition test using phenylboronic acid (PBA) and ethylenediaminetetraacetic acid (EDTA) [18]. A ≥ 5 mm difference in the inhibition zone between the imipenem disks without and with inhibitors (PBA, EDTA or both) was considered positive for KPC, metallo-β-lactamase or both carbapenemases. The presence of five common carbapenemase-encoding genes, *bla*_KPC_, *bla*_IMP_, *bla*_VIM_, *bla*_NDM_ and *bla*_OXA-48_, were genetically confirmed by PCR and DNA sequencing [19]. The final study sample consisted of 100 rectal swabs confirmed to be CPO-positive, and 100 rectal swabs with negative results were collected continuously.

### CPO Count of the Test Sample

The selected rectal swabs were eluted with 1 mL of normal saline. After vortex mixing, 100 µL of each sample was inoculated on CHROMagar KPC. Following incubation for 18 h at 37 ℃, the number of colonies was counted, and the number of bacteria recovered was expressed as colony forming units per millilitre (CFU/mL) [20].

### Xpert Carba-R Assay

Additionally, 100 µL of the above suspension was examined by Xpert Carba-R (Cepheid, Sunnyvale, CA, USA) according to the instructions. The CPO test results (negative or positive) and enzyme type were recorded. When the result of the Xpert Carba-R test was inconsistent with the expected result, the mixture from the original area on Columbia blood agar (Oxoid, Basingstoke, United Kingdom) was evaluated by Xpert Carba-R to confirm whether a CPO had been missed by the selective culture. Once a missed inspection of the CPO was confirmed, the result was modified to be CPO-positive and the relevant enzyme type was recorded.

### Broth enrichment and immunochromatographic assay

100 µL aliquots of the above suspension were added into five Eppendorf tubes, each containing 1 mL of lysogeny broth (LB) (Invitrogen, Carlsbad, CA, USA). The tubes were incubated with shaking at 200 rpm and 37 ℃ for 0 h, 1 h, 2 h, 3 h and 4 h respectively, and centrifuged at 12,000 rpm for 2 min, then the supernatant was discarded. The pellet was resuspended in the lysis buffer provided in the NG-Test CARBA 5 kit (four drops, 150 µL) and the sample was processed following the manufacturer’s instructions. When false-negative results were found, the CPO colonies that had been confirmed by PCR were directly evaluated with NG-Test CARBA 5 to exclude the negatives caused by the rare carbapenem enzyme type that cannot be detected by the NG-Test CARBA 5 test.

### Estimation of the limit of detection (LoD)

The LoD of NG-Test CARBA 5 after 4 h of broth incubation was estimated from 24 clinical CPO isolates (producing one or two of the carbapenemases KPC, NDM, IMP, VIM and OXA-48). A 0.5 McFarland turbidity standard suspension of each isolate was prepared and diluted to different concentrations by performing tenfold gradient dilutions with sterile normal saline. Additionally, equal amounts of faeces without a CPO were added to the same diluents to prepare a series of simulated faecal suspensions. One hundred microlitres of each saline suspension dilution and simulated faecal suspension was added to 1 mL of LB and cultured for 4 h. Then, the cultures were centrifuged, and the precipitates were assessed by NG-Test CARBA 5 as described above. The colony forming unit (CFU) titre of each bacterial suspension was determined by plating 100 µl of the above dilutions onto CHROMagar KPC and Columbia blood agar. The experiment was performed in double replicates, with the low value of each strain was taken as the estimated LoD.

### Statistical analysis

Data were analysed by SPSS v.19.0 software (SPSS Inc., Chicago, IL, USA). Sensitivity and specificity were determined by comparing the results of the Xpert Carba-R and broth enrichment and NG-Test CARBA 5 combined method with the expected results determined by PCR of the colonies or mixtures from the original area on Columbia blood agar. When calculating the total sensitivity and specificity, the overall result (positive or negative), but not the consistency of the enzyme type was considered. The McNemar test was used to compare whether the sensitivity and specificity of the combined method were better than those from Xpert Carba-R. A *p* value < 0.05 was considered statistically significant.

## Results

A total of 110 CPO isolates, which consisted of 57 isolates of *Klebsiella pneumoniae*, 34 isolates of *Escherichia coli*, 5 isolates of *Klebsiella oxytoca*, 5 isolates of *Enterobacter cloacae*, 4 isolates of *Citrobacter freundii*, 2 isolates of *Pseudomonas aeruginosa*, 1 isolate of *Pseudomonas monteilii*, 1 isolate of *Aeromonas caviae* and 1 isolate of *Acinetobacter baumannii*, were recovered from all 100 CPO-containing swabs. Molecular analysis confirmed that the samples contained 45 NDM, 41 KPC, 3 IMP, 1 VIM and 1 OXA-48 carbapenemases and 9 sampled harboured two or three carbapenemases simultaneously (5 NDM along with OXA-48, 2 NDM along with IMP, 1 KPC along with NDM, and 1 coharbouring KPC, NDM and OXA-48). Conventional phenotypic testing of carbapenemases through culture yielded results concordant with the molecular analysis for all isolates. Among the 100 samples that were negative by CHROMagar KPC screening, *bla*_NDM_ was detected by Xpert-Carba-R in two of them. Subsequently, the mixtures from the original area of Columbia blood agar were tested with Xpert-Carba-R, and the results were negative. The CPO count on CHROMagar KPC showed that 71.0% of the eluents of the positive swabs contained > 10^4^ CFU/mL, 22.0% contained 10^3^–10^4^ CFU/mL, and 7.0% contained < 10^3^ CFU/mL.

Xpert Carba-R demonstrated a combined 99.0% sensitivity and 98.0% specificity for the identification of CPO carbapenemase genes in rectal swabs. The sensitivity and specificity were higher than 90.0% for the different enzyme types (Table 1). However, this assay failed to detect the carbapenemase genes present in one specimen containing 80 CFU/mL NDM-producing *E. coli*. In addition, it should be noted that one or two more enzyme types were detected in 21 samples by the Xpert Carba-R assay, which did not contain relevant live bacteria. Among the 100 negative samples, the Xpert Carba-R test also showed 2 false-positives as described above.

**Table 1:** Performances of the Xpert Carba-R and NG-Test CARBA 5 in CPO screening of rectal swabs

Carbapenemase	Positive	Negative	NG-Test CARBA 5										Xpert Carba-R	
			0 h		1 h		2 h		3 h		4 h		Se	Sp
			Se	Sp	Se	Sp	Se	Sp	Se	Sp	Se	Sp		
KPC	43	157	34.9%	100%	51.2%	100%	72.1%	100%	86.0%	100%	93.0%	100%	97.7%	94.9%
NDM	54	146	33.3%	100%	53.7%	100%	70.4%	100%	96.3%	100%	96.3%	100%	98.1%	91.1%
IMP	5	195	0%	100%	0%	100%	40.0%	100%	100%	100%	100%	100%	100%	99.0%
VIM	1	199	0%	100%	100%	100%	100%	100%	100%	100%	100%	100%	100%	100%
OXA-48	7	193	42.9%	100%	42.9%	100%	42.9%	100%	85.7%	100%	85.7%	100%	100%	99.5%
Total^a^	100	100	34.0%	100%	53.0%	100%	72.0%	100%	93.0%	100%	96.0%	100%	99.0%	98.0%

*Abbreviations*: *KPC Klebsiella pneumoniae* carbapenemase, *NDM* New Delhi metallo-beta-lactamase, *IMP* imipenemase, *VIM* Verona integron-encoded metallo-beta-lactamase, *OXA* oxacillinase, *Se* Sensitivity, *Sp* Specificity

^a^When calculating the total sensitivity and specificity, the overall result (positive or negative), but not the consistency of the enzyme type was considered

The broth enrichment and NG-Test CARBA 5 combined method showed 96% sensitivity and 100% specificity after incubation for 4 h. A total of 93.0% of KPC (40/43), 96.3% of NDM (52/54), 100% of IMP (5/5), 100% of VIM (1/1), and 85.7% of OXA-48 (6/7) were detected with no false-positives. Of note, CPO was not detected in four specimens, which contained 10^3^–10^4^ CFU/mL KPC-producing *K. pneumoniae*, 10^3^–10^4^ CFU/mL NDM-producing *E. coli*, 80 CFU/mL NDM-producing *E. coli* and 30 CFU/mL KPC-producing *K. pneumoniae*. In one sample harbouring 10^3^–10^4^ CFU/mL NDM- and OXA-48-producing *E. coli*, this method missed detecting OXA-48. In another sample coharbouring > 10^4^ CFU/mL KPC-producing *K. pneumoniae* and > 10^4^ CFU/mL NDM- and OXA-48-producing *E. coli*, KPC was not detected. Compared with the Xpert Carba-R test, the specificity of the combined method was slightly higher (*p* = 0.50), but the sensitivity was slightly lower (*p* = 0.25); however, there were no significant differences (*p* > 0.05). After incubation for 3 h, the total sensitivity of this combined method was significantly lower than that of the Xpert Carba-R test (*p* = 0.03), while there was no significant difference in specificity (*p* = 0.50).

After 4 h broth incubation, the LoD of the NG-Test CARBA 5 assay for the different enzyme types varied from 7.5 × 10^2^ CFU/mL to 8.3 × 10^4^ CFU/mL, corresponding to 75 CFU/test and 8.3 × 10^3^ CFU/test, respectively, as shown in Table 2. In 2 of the 24 isolates, the LoD of the simulated faecal suspensions were higher than that of the bacterial suspensions. In addition, the LoDs of one carbapenemase were higher than that of the other in 2 of the 3 isolates that producing two carbapenemases.

**Table 2:** LoD determination of NG-Test CARBA 5 for different enzyme types after 4 h incubation

Carbapenemase type	Isolate	LoD			
		Bacterial Suspension		Simulated Fecal Suspension	
		CFU/mL	CFU/test	CFU/mL	CFU/test
KPC-2	*K. pneumoniae*	6.0 × 10^4^	6.0 × 10^3^	6.0 × 10^4^	6.0 × 10^3^
KPC-2	*K. pneumoniae*	4.2 × 10^4^	4.2 × 10^3^	4.2 × 10^4^	4.2 × 10^3^
KPC-2	*K. pneumoniae*	6.7 × 10^3^	6.7 × 10^2^	6.7 × 10^3^	6.7 × 10^2^
KPC-2	*K. pneumoniae*	7.1 × 10^3^	7.1 × 10^2^	7.1 × 10^3^	7.1 × 10^2^
KPC-2	*E. coli*	1.9 × 10^4^	1.9 × 10^3^	1.9 × 10^4^	1.9 × 10^3^
NDM-1	*K. pneumoniae*	6.5 × 10^3^	6.5 × 10^2^	6.5 × 10^3^	6.5 × 10^2^
NDM-1	*K. oxytoca*	1.0 × 10^4^	1.0 × 10^3^	1.0 × 10^4^	1.0 × 10^3^
NDM-1	*E. cloacae*	1.9 × 10^4^	1.9 × 10^3^	1.9 × 10^4^	1.9 × 10^3^
NDM-5	*E. coli*	8.0 × 10^3^	8.0 × 10^2^	8.0 × 10^3^	8.0 × 10^2^
NDM-5	*C. freundii*	1.2 × 10^4^	1.2 × 10^3^	1.2 × 10^4^	1.2 × 10^3^
IMP-4	*K. pneumoniae*	7.0 × 10^3^	7.0 × 10^2^	7.0 × 10^3^	7.0 × 10^2^
IMP-4	*K. oxytoca*	2.0 × 10^4^	2.0 × 10^3^	2.0 × 10^4^	2.0 × 10^3^
IMP-4	*E. cloacae*	1.9 × 10^4^	1.9 × 10^3^	1.9 × 10^4^	1.9 × 10^3^
IMP-8	*K. pneumoniae*	9.5 × 10^3^	9.5 × 10^2^	9.5 × 10^3^	9.5 × 10^2^
IMP-26	*E. cloacae*	1.7 × 10^4^	1.7 × 10^3^	1.7 × 10^4^	1.7 × 10^3^
VIM-1	*E. coli*	7.7 × 10^2^	77	7.7 × 10^3^	7.7 × 10^2^
VIM-1	*A. baumannii*	1.8 × 10^4^	1.8 × 10^3^	1.8 × 10^4^	1.8 × 10^3^
OXA-48	*K. pneumoniae*	1.3 × 10^4^	1.3 × 10^3^	1.3 × 10^4^	1.3 × 10^3^
OXA-48	*K. pneumoniae*	1.1 × 10^4^	1.1 × 10^3^	1.1 × 10^4^	1.1 × 10^3^
OXA-48	*K. pneumoniae*	6.5 × 10^3^	6.5 × 10^2^	6.5 × 10^3^	6.5 × 10^2^
OXA-48	*R. ornithinolytica*	7.5 × 10^2^	75	7.5 × 10^2^	75
KPC-2/ NDM-1	*K. oxytoca*	2.6 × 10^4^/2.6 × 10^3^	2.6 × 10^3^/2.6 × 10^2^	2.6 × 10^4^/2.6 × 10^3^	2.6 × 10^3^/2.6 × 10^2^
NDM-1/ IMP-4	*K. pneumoniae*	8.3 × 10^3^/8.3 × 10^3^	8.3 × 10^2^/8.3 × 10^2^	8.3 × 10^4^/8.3 × 10^4^	8.3 × 10^3^/8.3 × 10^3^
NDM-5/ OXA-181	*E. coli*	2.8 × 10^3^/2.8 × 10^4^	2.8 × 10^2^/2.8 × 10^3^	2.8 × 10^3^/2.8 × 10^4^	2.8 × 10^2^/2.8 × 10^3^

*Abbreviations*: *LoD* limit of detection, *KPC Klebsiella pneumoniae* carbapenemase, *NDM* New Delhi metallo-beta-lactamase, *IMP* imipenemase, *VIM* Verona integron-encoded metallo-beta-lactamase, *OXA* oxacillinase

## Discussion

The multiplex lateral flow immunochromatographic assay is a classical rapid detection method based on an antigen–antibody reaction. To date, several immunochromatographic assay kits have been developed to detect the five main carbapenemase families, KPC, NDM, IMP, VIM and OXA-48-like [11, 21–24]. Some of these kits can detect not only bacteria but also positive blood cultures directly [13, 14, 22, 25]. The performance of NG-Test CARBA 5 was proven to be good, with overall sensitivity and specificity values that ranged from 92.1 to 100% and 95.3 to 100%, respectively [15, 24, 26–28].

The broth enrichment and multiplex lateral flow immunochromatographic assay combined method used for the rapid detection of CPOs in rectal swabs has rarely been reported [11, 16, 29]. Compared with PCR, the sensitivity of NG-Test CARBA 5 for the detection of KPC, VIM, and NDM was 80.0% without incubation and 88.0% with one hour, 92.0% with two hours, and 100% with three hours incubation, while the specificity was 100% at all time points [16]. However, the limitations of the study were that the number of samples analysed was small (*n* = 20), and the tests for IMP and OXA-48 were not evaluated due to a lack of positive strains. Thus, expanded research is needed. Another study showed the performance of the OKN K-SeT test (Coris BioConcept, Gembloux, Belgium) for the rapid detection of OXA-48, KPC and NDM carbapenemase-producing Enterobacterales directly from rectal swab samples, with an overall sensitivity of 96.0% and a specificity of 100% [11]. However, since the OKN K-SeT kit is not precoated with IMP or VIM antibodies, IMP and VIM CPOs cannot be detected. To the best of our knowledge, the distribution of carbapenemase genes vary according to region [30–33]. KPC, NDM, IMP, VIM, and OXA-48 are the most prevalent enzymes in Enterobacterales in China and some other countries [30, 34, 35]. Screening rectal swabs for CPOs with a kit targeting all five carbapenemases at once, such as NG-Test CARBA 5, has more clinical application value and can be commercialized.

In this study, the results from various CPO detection methods were comprehensively considered to determine the true results (i.e., the expected results). Overall, a total of 200 samples were included, including 100 CPO-positive and 100 CPO-negative rectal swabs. These samples covered all five enzymes that can be detected by NG-Test CARBA 5, as well as common CPO species. The results showed that the total sensitivity and specificity of the broth enrichment NG-Test CARBA 5 combined method were 93.0% and 100% when incubation was carried out for 3 h and 96.0% and 100% after 4 h of incubation, respectively. Comparing the combined method with Xpert Carba-R, there was no significant difference in the total sensitivity after incubation for 4 h (*p* = 0.25) but there was a significant difference after 3 h (*p* = 0.03). The total sensitivity of the combined method was significantly lower than that of the Xpert Carba-R test when incubated for 3 h. Moreover, there were no significant differences in total specificity after different incubation times (*p* = 0.50). This result suggested that the detection performance of NG-Test CARBA 5 was equivalent to that of Xpert Carba-R after 4 h broth incubation. Although this method is not as fast or as simple as molecular detection methods, it is less expensive and does not require special instrumentation. This method can thus be used as an alternative to molecular detection methods for CPO screening in economically underdeveloped areas or hospitals lacking molecular detection instruments.

The LoD of NG-Test CARBA 5 after 4 h broth incubation was evaluated. The data showed that the LoD was between 10^2^ and 10^4^ CFU/mL for the different carbapenemases. Since the actual CPO content in some specimens was equal to or lower than the LoD, NG-Test CARBA 5 failed to detect CPO in some cases. In addition, it should be noted that the expression of different enzymes was different in the strain producing more than one carbapenemase. Additionally, different enzymes showed competitive inhibition in the multiplex lateral flow immunochromatographic assay, and some enzyme types were not detected in some of the samples containing multiple enzymes. Comparing the LoDs of the simulated faecal suspension with those of the bacterial suspension, it was noted that faeces slightly interfered with the detection of carbapenemases in some cases. The influence of different components of faeces, such as the presence of other bacteria, on carbapenemase detection requires further evaluation.

We also noticed that one or two more enzyme types were detected in 21 of the 100 positive samples by the Xpert Carba-R assay without evidence of living bacteria. Among the 100 negative samples, the Xpert Carba-R test showed 2 false-positives. This result suggested that the PCR-positive signals might be a result of the amplification of DNA fragments that remained in the samples. The report by Fauconnier et al.’s also mentioned this point in the discussion [11]. The sensitivity of the Xpert Carba-R test in CPO screening of rectal swabs was slightly higher, but the specificity was slightly lower than that of NG-Test CARBA 5 after 4 h of incubation. However, the difference was not statistically significant (*p* > 0.05). In general, the sensitivity and specificity of both methods were higher than 95%, which is acceptable for CPO screening.

Although various details affecting the accuracy of the results have been considered, there are also limitations in this study. During the whole study period, relatively few IMP, VIM and OXA-48 positive swabs were collected. To evaluate the detection performance of NG-Test CARBA 5 after different incubation times, rectal swabs were eluted with 1 mL of normal saline to meet the different detection needs with the same sample size, and did not fully represent clinical samples after tenfold dilutions. Instead, colony counting was performed and the CPO content of each swab was calculated. We noticed that the bacterial counts of the false-negative samples were all near the LoD. It has been suggested that CPO detection rate is affected by sampling and can be improved by increasing the amount of stool. Therefore, stool samples are better than rectal swab samples when only the sample content is considered.

## Conclusions

This comparative study of NG-Test CARBA 5 versus Xpert Carba-R that detected CPOs in rectal swabs showed that after 4 h of broth incubation, NG-Test CARBA 5 had an equivalent detection capability to Xpert Carba-R. The broth enrichment and NG-Test CARBA 5 combined method is recommended as a new scheme for CPO screening as it is less expensive, although Xpert Carba-R is less laborious and faster.

## Supplementary Information

**Additional file 1.**

## Data Availability

The original contributions presented in the study are included in the article/supplementary material, further inquiries can be directed to the corresponding authors.

## Abbreviations

CPO: Carbapenemase-producing organism
KPC: *Klebsiella pneumoniae* carbapenemase
NDM: New Delhi metallo-beta-lactamase
IMP: Imipenemase
VIM: Verona integron-encoded metallo-beta-lactamase
OXA: Oxacillinase
PCR: Polymerase chain reaction
CLSI: Clinical and Laboratory Standards Institute
PBA: Phenylboronic acid
EDTA: Ethylenediaminetetraacetic acid
LB: Lysogeny broth
LoD: Limit of detection
CFU: Colony forming units
Se: Sensitivity
Sp: Specificity
